# Bcr/Abl Interferes with the Fanconi Anemia/BRCA Pathway: Implications in the Chromosomal Instability of Chronic Myeloid Leukemia Cells

**DOI:** 10.1371/journal.pone.0015525

**Published:** 2010-12-28

**Authors:** Antonio Valeri, Maria Eugenia Alonso-Ferrero, Paula Río, María Roser Pujol, José A. Casado, Laura Pérez, Ariana Jacome, Xabier Agirre, Maria José Calasanz, Helmut Hanenberg, Jordi Surrallés, Felipe Prosper, Beatriz Albella, Juan A. Bueren

**Affiliations:** 1 Centro de Investigaciones Energéticas, Medioambientales y Tecnológicas (CIEMAT) and Centro de Investigación Biomédica en Red de Enfermedades Raras (CIBERER), Madrid, Spain; 2 Departamento de Genética y Microbiologia, Universitat Autònoma de Barcelona, Bellaterra, Spain; 3 Centro de Investigación Biomédica en Red de Enfermedades Raras (CIBERER), Barcelona, Spain; 4 Fundación para la Investigación Médica Aplicada (CIMA), Clínica Universidad de Navarra, Pamplona, Spain; 5 Department of Pediatric Oncology, Hematology and Immunology, Children's Hospital, Duesseldorf, Germany; 6 Department of Pediatrics, Wells Center for Pediatric Research, Riley Hospital for Children, Indianapolis, Indiana, United States of America; St Georges University of London, United Kingdom

## Abstract

Chronic myeloid leukemia (CML) is a malignant clonal disorder of the hematopoietic system caused by the expression of the *BCR/ABL* fusion oncogene. Although it is well known that CML cells are genetically unstable, the mechanisms accounting for this genomic instability are still poorly understood. Because the Fanconi anemia (FA) pathway is believed to control several mechanisms of DNA repair, we investigated whether this pathway was disrupted in CML cells. Our data show that CML cells have a defective capacity to generate FANCD2 nuclear foci, either in dividing cells or after DNA damage. Similarly, human cord blood CD34^+^ cells transduced with *BCR/ABL* retroviral vectors showed impaired FANCD2 foci formation, whereas FANCD2 monoubiquitination in these cells was unaffected. Soon after the transduction of CD34^+^ cells with *BCR/ABL* retroviral vectors a high proportion of cells with supernumerary centrosomes was observed. Similarly, *BCR/ABL* induced a high proportion of chromosomal abnormalities, while mediated a cell survival advantage after exposure to DNA cross-linking agents. Significantly, both the impaired formation of FANCD2 nuclear foci, and also the predisposition of *BCR/ABL* cells to develop centrosomal and chromosomal aberrations were reverted by the ectopic expression of *BRCA1*. Taken together, our data show for the first time a disruption of the FA/BRCA pathway in *BCR/ABL* cells, suggesting that this defective pathway should play an important role in the genomic instability of CML by the co-occurrence of centrosomal amplification and DNA repair deficiencies.

## Introduction

Chronic Myeloid Leukemia (CML) is a clonal hematopoietic disorder generated by a t(9;22)(q34;q11) translocation resulting in a *BCR/ABL* oncogene[1], [2] that encodes for a tyrosine kinase BCR/ABL-p210 oncoprotein[3]. Although several genetic defects are accumulated in CML cells during the progression from the chronic phase towards the accelerated and blast crisis phases (see review in [4]), studies in mice transplanted with *BCR/ABL* transduced cells demonstrated that this oncogene is the causative agent of CML[5].

In addition to a differentiation arrest, failures in the genomic surveillance and DNA repair of CML cells account for the natural malignant progression of the disease (see review in[6]). Although the mechanisms by which BCR/ABL interferes with the genomic stability of the cell are still poorly understood, the effects of this oncoprotein upon DNA damage, apoptosis and DNA repair are considered critical processes facilitating the accumulation of mutations during the progress to blast crisis (see review in [7]). Moreover, increasing evidence has been published showing that BCR/ABL induces reactive oxygen species (ROSs) causing oxidative damage to CML cells[8], and therefore a variety of DNA lesions, including the highly mutagenic double strand breaks (DSBs)[9], [10]. These effects, together with the reported effect of this oncoprotein on the efficacy and/or the fidelity of different DNA repair mechanisms[9], [11], [12] contribute to explain the mutator phenotype of CML cells.

Concerning the mechanisms by which BCR/ABL affects the repair of the DSBs, previous studies have shown that this oncoprotein interferes both with the non-homologous end joining (NHEJ) pathway and with pathways that utilize homologous templates. Regarding the effects of BCR/ABL on classic NHEJ, Deutsch *et al* observed that the catalytic subunit of DNA-dependent protein kinase (DNA-PKcs), a key protein in this major DNA repair system in mammalians, was down-regulated in CML cells[13]. In addition to NHEJ, BCR/ABL has also been involved in the aberrant regulation of the two pathways that utilize homologous templates, the faithful homology directed repair (HDR) and the mutagenic single strand annealing (SSA). Interestingly, previous studies have shown that BRCA1, a critical protein for preserving the genomic integrity by promoting homologous recombination[14], is nearly undetectable in CML cells[15]. On the other hand, more recent studies have shown that BCR/ABL specifically promotes the repair of DSBs through SSA, a mutagenic pathway that involve sequence repeats[16], [17].

Because the Fanconi anemia (FA) pathway is believed to control several DNA repair pathways, and therefore the genomic stability of the cell (see review in[18]), we ought to investigate the integrity of this pathway in CML cells. Thirteen FA proteins have been identified in the FA pathway, each of them participating in one of the three FA protein complexes. The upstream complex – the FA core complex - is integrated by eight FA proteins (FANCA, FANCB, FANCC, FANCE, FANCF, FANCG, FANCL, FANCM) and two FA associated proteins (FAAP24 and FAAP100). A second complex is formed by FANCD2 and FANCI, which work together in the FA-ID complex. Because of the E3 ligase activity (FANCL) of the FA core complex, FANCD2 and FANCI can be monoubiquitinated and then loaded onto chromatin, forming large nuclear foci in response to DNA damage or replication arrest. Finally, monoubiquitinated FANCD2/FANCI interact with downstream FA proteins such as FANCJ/BRIP1, FANCN/PALB2 and FANCD1/BRCA2, which form stable complexes with proteins participating in HDR, like BRCA1 and RAD51[19], [20].

The results presented in this study demonstrate for the first time that CML cells are characterized by a defective FA/BRCA pathway, downstream FANCD2 monoubiquitination. In particular we demonstrate that BCR/ABL interferes with the formation of nuclear FANCD2 foci, in a process that can be reverted by the ectopic expression of *BRCA1*. The consequences of this defect upon the genetic stability of *BCR/ABL* cells are shown.

## Materials and Methods

### Culture of CD34^+^ cells from CML patients and healthy umbilical cord blood

Studies were approved by the authors' Institutional Review Board and conducted under the Declaration of Helsinki rules. Chronic myeloid leukemia CD34^+^ cells were obtained from the peripheral blood (PB) of CML patients, after previous informed consents approved by the ethical Committee of the Clínica Universitaria de Navarra. Healthy CD34^+^ cells were obtained from umbilical cord bloods (CB) scheduled for discard, after written informed consents of the mother. Mononuclear cells (MNCs) were obtained by fractionation in Ficoll-hypaque according to manufacturer’s instructions (GE Healthcare, Stockholm, Sweden). Purified CD34^+^ cells were obtained using a MACS CD34 Micro-Bead kit (Miltenyi Biotec, Gladbach, Germany). For the expansion of CML CD34^+^ cells, samples were cultured in StemSpan medium (Stem Cell Technologies, Vancouver, BC, Canada) supplemented with 100 ng/ml human stem cell factor (hSCF; Peprotech, London, UK), 100 ng/ml Flt3-L (Invitrogen, Carlsbad, CA), 20 ng/ml IL-6 (Peprotech) and 20 ng/ml IL-3 (Biosource).

### Cell lines

Human-derived Mo7e (a megakaryoblastic leukaemia cell line without the *BCR/ABL* fusion) and Mo7e-p210 cells (Mo7e cells transfected with p210 isoform of *BCR-ABL1*) [21], [22] were cultured in RPMI medium (Gibco, NY) supplemented with 10% fetal bovine serum (FBS; Lonza, Belgium), 2 mM L-glutamine (Gibco), 100 U/mL penicillin/streptomycin (Gibco) at 37°C in a humidified atmosphere with 5% CO_2_. Mo7e cells were grown in medium supplemented with 10 ng/ml of hr-IL3 (Biosource). FA-A LCLs were cultured in RPMI medium (Gibco) supplemented with 15% FBS, 2 mM L-glutamine (Gibco), 100 U/mL penicillin/streptomycin (Gibco) at 37°C in a humidified atmosphere with 5% CO_2_.

### Retroviral vectors production

The control retroviral vector (RV) used in these experiments was the MIG-R1 retroviral vector which consist on a MSCV-IRES-GFP vector. The MIG-210 is derived from the MIG-R1, and contains the full-length b3a2 *BCR/ABL* cDNA under the control of the MSCV promoter. Both vectors were kindly provided by Bryan G. Druker (Oregon Health and Science University, Portland, OR). Where indicated, cells were transduced with similar RVs (MIN-210 and its respective MIN-R1 control, kindly provided by W. S. Pear). In these vectors, a truncated version of the NGFR (*Δ*NGFR) cDNA was used instead of the EGFP marker gene, to facilitate the immunoselection of transduced cells. In order to ectopically express BRCA1 in control and BCR/ABL positive cells, S11Brca1-IRES-Neo and S11-IRES-Neo RVs were generated. Retroviral vectors were produced and titrated as previously described[23].

### Retroviral transduction of hematopoietic progenitors from human cord blood

Human CB CD34^+^ cells were pre-stimulated for 48 h with StemSpan (StemCell technologies) supplemented with 300 ng/ml hSCF (Peprotech), 100 ng/ml hTPO (R&DSystems, Minneapolis, MN) and 100 ng/ml Flt3-L (Invitrogen, Carlsbad, CA). Pre-stimulated cells were re-suspended at a density of 5×10^5^ cells/ml in retroviral supernatant medium supplemented with FBS (20% final concentration) and growth factors. Cells were then added to retronectin-coated wells (Retronectin, Takara Shuzo, Otsu, Japan) preloaded with the correspondent retroviral vector. Supernatants were replaced every 12 h by new virus containing medium. A total of four transduction cycles were routinely conducted[23].

When hCB CD34^+^ cells were transduced with MIN-R1 and MIN-210 and purified by immunomagnetic cell sorting with anti-NGFR beads (Miltenyi Biotech) 2 days after transduction following manufacturer's instructions. Purified populations contained at least 95% of NGFR^+^ cells.

### DNA damage treatments and drugs exposure

To conduct Western Blotting and Immunofluorescence assays, cells were treated with Imatinib 1 µM (Novartis Pharma, Basel, Switzerland) for 24 hours and then treated with 40 nM of mitomycin C (MMC) for 16 hours. For inhibition studies cells were treated for 16 hours with MMC and afterwards 1 hour with 40 µM of MG132 (Sigma-Aldrich, ST.Louis, MO) or 20 µM of LY294002 (Cell Signaling Technology, Inc, Danvers, MA). To test the cellular resistance to MMC, clonogenic assays were conducted in semisolid medium (Methocult H4434, StemCell Technologies) containing increasing concentrations of the drug, plated in triplicate on 35-mm plastic culture dishes (Nunc, Roskilde, Denmark) and cultured at 37°C, 5% CO_2_ and fully humidified air. Fourteen days after plating, the total number of colonies was scored under an inverted microscope.

### Chromosomal breakage analyses

CB CD34^+^ cells were transduced with MIN-210 or its respective MIN-R1 control and purified two days afterwards, prior to re-infect these samples with *Neo^r^* or *BRCA1/Neo^r^* vectors. Samples were left untreated or treated with 0.1 µg/ml of diepoxibutane (DEB) for a 72-h period. To obtain metaphases, colcemid (0.1 µg/ml; Gibco) was added 24 h prior to harvesting. Cells were then treated with hypotonic solution (0.075 M KCl, Sigma), fixed in methanol:acetic acid (3∶1 vol/vol), dropped onto clean slides and air-dried O.N. following standard cytogenetic procedures. Slides were stained with 10% Giemsa in phosphate buffer, pH 6.8. Fifty to seventy metaphases per sample were analyzed for chromosome aberrations including gaps, chromosome and chromatid breaks, acentric fragments, and chromosome- and chromatid-type exchanges.

### Flow cytometry analyses

For cell cycle analyses, aliquots of 10^5^ cells were washed twice in PBS and fixed in 4.5 mL ice-cold 1% methanol-free formaldehyde in PBS for 15 min on ice. After centrifugation, 5 mL of 70% ethanol (0–4°C) was added, and cells were stored at −20°C at least for 2 hours. After washing, cells were resuspended in 1 mL propidium iodide solution (5 µg/mL; Molecular Probes) with 100 µg/mL DNase-free RNase A (Sigma) and incubated for 30 min at 37°C. Finally, cells were analyzed by flow cytometry (Coulter XL) with linear fluorescence of propidium iodide (DNA content) from 10,000 events with doublet discrimination.

### Western Blot analyses

Western blot analyses were performed using extracts of purified CD34^+^ cells collected by centrifugation. After electrophoresis, proteins were transferred to a nitrocellulose membrane. After blocking, the membrane was incubated at 4°C O.N. with the primary antibodies (anti-FANCD2, Abcam ab2187) diluted in blocking solution, washed extensively and incubated with the appropriate secondary antibody using the Western Breeze Immunodetection Kit (Invitrogen), according to methods previously described[24].

### Immunofluorescence studies

In these studies cells were fixed with 3.7% paraformaldehyde in PBS for 15 minutes followed by permeabilization with 0.5% Triton X-100 in PBS for 5 min. After blocking for 30 minutes in Blocking buffer (10% FBS, 0.1% NP-40 in PBS) cells were incubated with rabbit polyclonal anti-FANCD2 (Abcam, ab2187-50), mouse monoclonal anti-γH2AX (Upstate, JBW301), and mouse monoclonal anti-BRCA1 (Oncogene, ab-1 and ab-3). For centrosomal staining, cells were fixed and permeabilized and incubated with primary antibody against γ-tubulin (Abcam, ab16504). Cells were subsequently washed three times in TBS (50 mM Tris-HCl (pH 8.0), 150 mM NaCl) and incubated with anti-mouse or anti-rabbit Texas Red (Molecular Probes, Leiden, The Netherlands) as secondary antibodies. After 45 min. cells were washed three times with TBS and the slides were mounted in Moviol with 4,6-diamidino-2 phenylindole (DAPI). Slides were analyzed with a fluorescence microscope Axioplan2 (Carl Zeiss, Göttingen, Germany) using a 100x/1.45 oil working distance 0.17-mm objective. The proportion of cells with nuclear foci was scored as described elsewhere[24] after analyzing 200 cells per slide. Immunofluorescent images were acquired with an AxioCam MRm (Carl Zeiss) and were processed with AxioVision version 4.6.3 (Carl Zeiss) and Corel Photo-Paint 11 (Corel, Ottawa, Canada).

### Statistical analysis

Results are shown as the mean±s.e. Differences between groups were assessed using the two tailed Student’s t-test. The data from the clonogenic assays were calculated as survival percentages respect to control cultures. The survival data were fitted by least squares only for experiments with at least three available data points. The IC50 value was obtained algebraically, solving the fitted quadratic equation for the value of dose where the estimated percentage of surviving cells would equal 50%. The processing and statistical analysis of the data was performed by using the Statgraphics Plus 5.0 software package (Manugistics, Inc. Rockville, MD).

## Results

### BCR/ABL interferes with the formation of FANCD2 nuclear foci in hematopoietic progenitors from chronic myeloid leukemia patients

**Figure 1:**
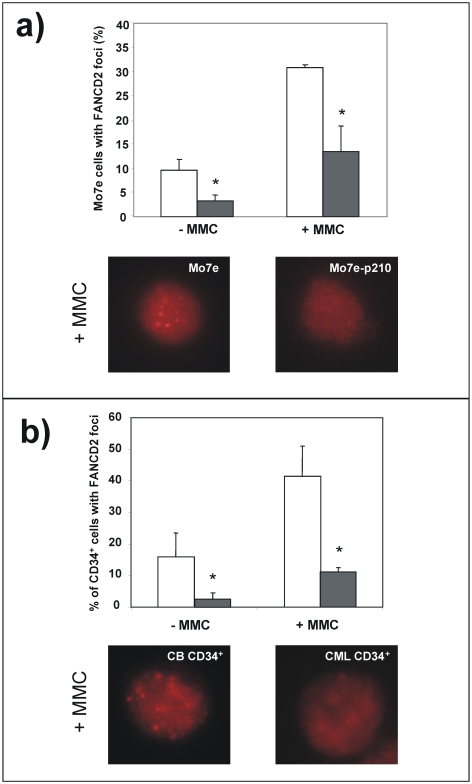
Impaired formation of nuclear FANCD2 foci in Mo7e-p210 and CD34^+^ cells from CML patients. a) Analysis of the proportion of Mo7e (white bars) and Mo7e-210 cells (grey bars) with FANCD2 nuclear foci, either untreated or treated with MMC (40 nM; 16 h). Panel b) shows the proportion of CD34^+^ cells from healthy cord blood (white bars) or from the peripheral blood of CML patients (grey bars) with nuclear FANCD2 foci. Bars show mean ± s.e. of values corresponding to three independent experiments. *Differences between healthy and CML CD34^+^ cells were significant at p<0.01. Representative pictures of nuclear FANCD2 foci in MMC-treated samples are shown.

Because of the relevance of the FA/BRCA pathway in the control of DNA repair, we first investigated whether CML cells had a disruption in this pathway. To this aim, and given that the loading of FANCD2 to chromatin constitutes a central process in the FA/BRCA pathway[20], we determined the proportion of cells with nuclear FANCD2 foci, both in a cell line stably transfected with the *BCR/ABL* oncogene (Mo7e-p210 cells) and in the control parental cells (Mo7e cells). As shown in Figure 1a, the proportion of Mo7e-p210 cells with FANCD2 nuclear foci was significantly reduced compared to control Mo7e cells. This effect was evident in samples not exposed to any DNA damaging agent, and also in cells treated with the DNA cross-linking drug MMC.

To investigate whether data obtained in Mo7e-p210 cells was reproduced in primary CML cells, similar studies were conducted with peripheral blood (PB) CD34^+^ cells from CML patients at diagnosis, and from healthy CD34^+^ cells obtained from cord blood (CB) samples. Similar to Mo7e cells, the proportion of CD34^+^ cells with FANCD2 foci was significantly reduced when samples were obtained from CML patients, compared to healthy CD34^+^ cells. As in Mo7e cells, differences between both groups were significant, both in untreated and in MMC-treated cells (Figure 1b).

**Figure 2:**
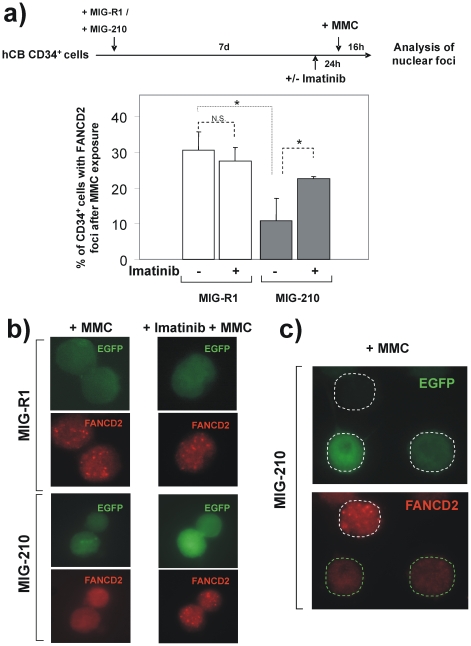
Direct effect of *BCR/ABL* upon the formation of nuclear FANCD2 foci in human cord blood CD34^+^ cells. a) Experimental protocol and analysis of the proportion of cord blood CD34^+^ cells with FANCD2 nuclear foci after transduction with retroviral vectors expressing *EGFP* (MIG-R1) or *EGFP* plus *BCR/ABL* (MIG-210). The effect of imatinib upon the formation of FANCD2 foci in these cells is also shown. In all instances cells were treated with MMC (40 nM) 16 h prior to conduct the immunofluorescence studies. Bars show mean ± s.e. of values corresponding to three independent experiments. *Differences were significant at p<0.01. b) Representative pictures showing restored formation of FANCD2 nuclear foci in MIG-210-transduced CD34^+^ cells treated with imatinib. Nuclear foci were exclusively scored in cells expressing the retroviral vector marker gene (EGFP). c) Representative pictures showing the specific inhibition of FANCD2 foci in human CD34^+^ cells transduced with MIG-210. Note that only cells expressing the marker EGFP, co-expressed with BCR/ABL in this vector, are negative for FANCD2 foci.

The inhibition of FANCD2 foci in Mo7e-p210 cells and in primary CML cells could result from either a direct effect of the *BCR/ABL* oncogene, or through accumulated mutations that may have occurred along the culture of the cell line or during the progression of the disease of CML patients. To understand whether this effect was directly generated by the *BCR/ABL* oncogene, CB CD34^+^ cells were transduced with a control MIG-R1 vector (only expressing the *EGFP* marker gene) or with the oncogenic MIG-210 vector (expressing both the *BCR/ABL* and the *EGFP* genes), and exposed to MMC seven days afterwards. Additionally, one aliquot of these samples was incubated with imatinib 24 h prior to MMC treatment, to evaluate whether potential effects mediated by BCR/ABL were dependent on the tyrosine kinase activity of the oncoprotein (See schematic experimental protocol in Figure 2a). To ensure that FANCD2 foci were scored exclusively in cells that had been transduced with either MIG-R1 or MIG-210 vectors, only green fluorescent cells were considered for the analysis of nuclear FANCD2 foci.

Consistent with the results in Figure 1, the proportion of MMC-treated CD34^+^ cells with FANCD2 foci was significantly inhibited when samples were transduced with MIG-210, as compared to the control MIG-R1 vector (Figure 2a). Moreover, while imatinib did not significantly affect the formation of nuclear FANCD2 foci in cells transduced with the control vector, this drug significantly increased the proportion of MIG-210 transduced CD34^+^ cells with FANCD2 foci (see Figure 2a and representative pictures in Figure 2b). The effect of BCR/ABL upon the formation of nuclear FANCD2 foci was confirmed in MIG-210-transduced CD34^+^ cells, by the observation that only EGFP-negative (untransduced) cells contained evident nuclear FANCD2 foci; while EGFP fluorescent cells (thus harbouring the MIG-210 provirus) were mostly negative for FANCD2 foci (see representative pictures in Figure 2c). These results indicate that the tyrosine kinase activity of p210 is responsible of the impaired formation of nuclear FANCD2 foci in MIG-210 transduced CD34^+^ cells.

Because the decreased proportion of MIG-210 transduced CD34^+^ cells with FANCD2 foci could be consequence of a reduced number of cells in S-phase[25], we investigated the cell cycle distribution of MIG-R1 and MIG-210 transduced cells. As shown in Figure S1, the proportion of CD34^+^ cells in S-phase was increased, rather than decreased, after transduction with the MIG-210 vector. Additionally, and given that FA cells are classically arrested in the G2/M phase after treatment with DNA cross-linking drugs[26], we also determined the percentage of MIG-210-transduced CD34^+^ cells in G2/M after MMC exposure. As shown in representative histograms of Figure S1, no evident blockage in the G2/M phase was observed in MMC-treated MIG-210-transduced CD34^+^ cells at doses conventionally used for FA diagnosis (40 nM).

We also speculated that the inhibitory effects of BCR/ABL upon the formation of FANCD2 foci in MMC-treated cells could be due to a potentially lower generation of DSBs in *BCR/ABL* cells. To rule out this possibility, we determined the generation of DSBs in MIG-R1 and MIG-210 transduced CB CD34^+^ cells by means of the analysis of γ-H2AX nuclear foci. A higher number of DSBs was observed in MIG-210 cells compared to MIG-R1 cells in the absence of MMC treatment (Figure S2), consistent with the reported effects of BCR/ABL on the generation of ROSs[8], [9], [10]. After MMC, a similar proportion of cells with γ-H2AX foci was observed in control and p210-transduced cells (Figure S2). These observations indicate that the low proportion of *BCR/ABL* cells with FANCD2 foci is not attributable to an impaired generation of DSBs.

### The impaired formation of nuclear FANCD2 foci in *BCR/ABL* cells is not due to a defect in FANCD2 monoubiquitination

**Figure 3:**
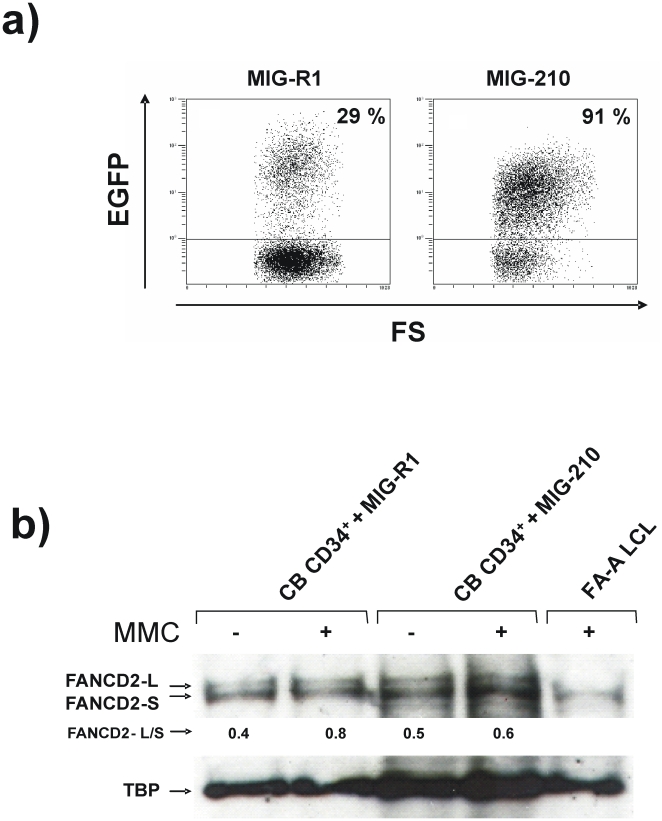
Efficient monoubiquitination of FANCD2 in *BCR/ABL*- transduced cord blood CD34^+^ cells. a) Flow cytometry analysis showing the proportion of cord blood CD34^+^ cells expressing the retroviral marker EGFP, 7 days after transduction with MIG-R1 or MIG-210 vectors. b) Western blot analysis of monoubiquitinated (FANCD2-L) and non ubiquitinated FANCD2 (FANCD2-S) in samples shown in panel a. As a negative control of FANCD2 ubiquitination, LCLs from a FA-A patient was also included. Ratios between FANCD2-L/FANCD2-S are shown.

Since the formation of nuclear FANCD2 foci requires the monoubiquitination of this protein[27], we then investigated whether *BCR/ABL* was able to inhibit FANCD2 monoubiquitination in CB CD34^+^ cells. Although MIG-R1 and MIG-210 vectors mediated similar transductions of CB CD34^+^ cells (28% and 32% of CD34^+^ cells were EGFP^+^, respectively, at 48 h post-transduction), 91% of MIG-210 transduced cells expressed the EGFP marker 7 days after transduction, while 29% of MIG-R1 transduced cells were EGFP^+^ at this time (Figure 3a), showing that *BCR/ABL* mediates a proliferation advantage in CD34^+^ cells. Nuclear protein extracts from these samples were analyzed by western blot to investigate the presence of the monoubiquitinated and non-ubiquitinated FANCD2 bands. As expected, the negative control consisting of FA-A LCLs only expressed the non-ubiquitinated FANCD2 isoform (FANCD2-S). FANCD2 was, however, efficiently monoubiquitinated (FANCD2-L) not only in control CD34^+^ cells transduced with the MIG-R1 vector, but also in MIG-210 transduced CD34^+^ cells (see Figure 3b). These results thus indicate that BCR/ABL does not interfere with the function of the FA core complex, required for FANCD2 monoubiquitination, but rather with downstream steps in the FA pathway.

### The impaired formation of FANCD2 foci in *BCR/ABL* cells can be reverted by inhibitors of the proteasome and the PI3K/Akt pathway and by the ectopic expression of BRCA1

**Figure 4:**
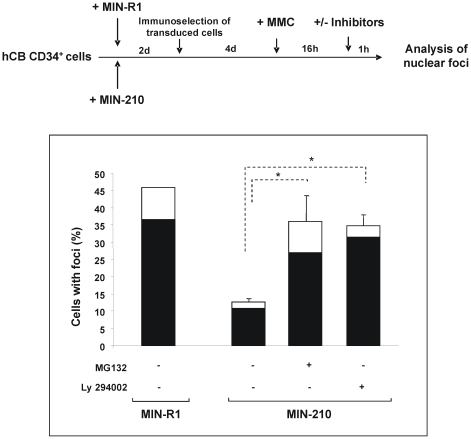
Reversion of the deficient formation of BRCA1 and FANCD2 foci in *BCR/ABL*-transduced cord blood CD34^+^ cells by inhibitors of the proteasome and the PI3K/Akt pathway. Experimental protocol and analysis of the proportion of MIN-R1 and MIN-210-transduced cord blood CD34^+^ cells with BRCA1 (white bars) or FANCD2 foci (black bars) after treatment with the proteasome inhibitor MG132, or the PI3K/AKT inhibitor Ly294002. Bars show mean ± s.e. of values corresponding to three independent experiments. *Differences were significant at p<0.01.

Previous studies in healthy cells have shown that BRCA1 is required for the accumulation of FANCD2 at sites of DNA damage but not for FANCD2 monoubiquitination[27], [28], [29]. Given that BRCA1 levels are decreased in *BCR/ABL* cells[15], we investigated the involvement of BRCA1 in the defective capacity of *BCR/ABL* cells to generate FANCD2 foci, using two different pharmacological approaches. Because of the involvement of proteasome in reduced BRCA1 levels observed in *BCR/ABL* cells [15], the effect of a proteasome inhibitor, MG132, upon the formation of BRCA1 and FANCD2 foci was first investigated. Additionally, because the PI3K/AKT chemical inhibitor, LY294002, has been described to control BRCA1 activation in breast cancer cells [30], this inhibitor was also used in parallel to MG132. Purified MIN-210 and MIN-R1 transduced CD34^+^ cells were treated with MMC for 16 h, and then with MG132 or LY294002 prior to conduct immunofluorescence analyses of BRCA1 and FANCD2 foci (Figure 4). As shown in this figure, the proportion of *BCR/ABL* cells with nuclear BRCA1 foci, and also of FANCD2 foci, was significantly increased when samples were treated with either of these inhibitors.

**Figure 5:**
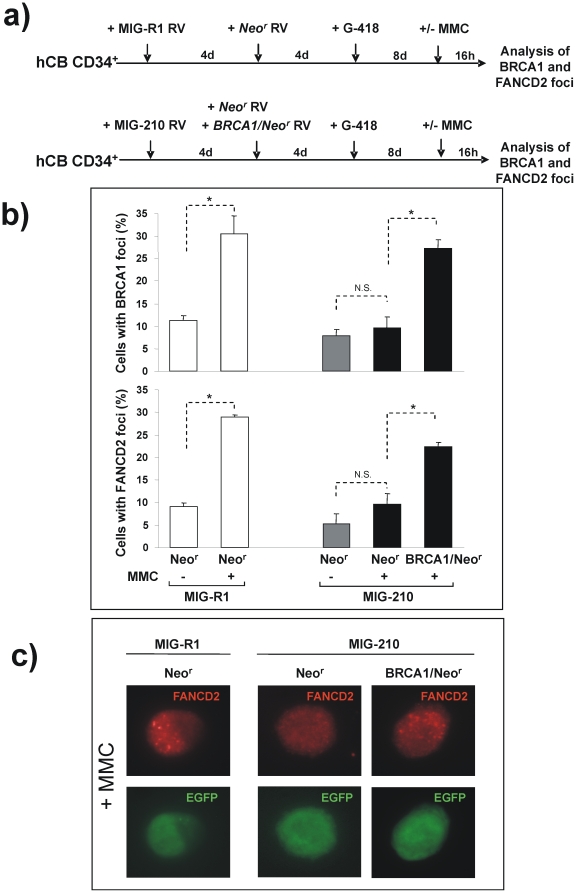
Reversion of the deficient formation of BRCA1 and FANCD2 foci in *BCR/ABL*-transduced cord blood CD34^+^ cells by the ectopic expression of *BRCA1*. a) Experimental protocol used for investigating the effects mediated by the ectopic expression of BRCA1 upon the formation of BRCA1 and FANCD2 foci in *BCR/ABL-*transduced cells. b) Analysis of the proportion of MIG-R1 (white bars) and MIG-210 (grey and black bars) transduced cord blood CD34^+^ cells with BRCA1 or FANCD2 foci after re-infection with vectors expressing the phosphotransferase gene (Neo^r^; grey bars) or *BRCA1* plus *neo^r^* (BRCA1/Neo^r^; black bars). Samples were exposed to 0 or 40 nM MMC prior to analyses of nuclear foci in EGFP^+^ cells. Bars show mean ± s.e. of values corresponding to three independent experiments. *Differences were significant at p<0.01. c) Representative pictures of MMC-treated cells corresponding to panel b, are shown.

Since MG132 and LY294002 may have effects upon pathways not directly related to BRCA1, we aimed to confirm the role of BRCA1 in the impaired formation of FANCD2 foci of *BCR/ABL* cells by means of the ectopic expression of BRCA1 in these cells. To this aim, CD34^+^ cells transduced with MIG-R1 and MIG-210 retroviral vectors were re-transduced four days afterwards with *neo^r^* or *BRCA1/neo^r^* retroviral vectors. Co-transduced cells were then selected with geneticin for eight days, and finally exposed to MMC or maintained in MMC-free medium. Geneticin-resistant cells that expressed the EGFP protein, thus co-transduced with *BCR/ABL* and *BRCA1* vectors or their respective controls, were scored for the formation of FANCD2 and BRCA1 foci (see schematic protocol in Figure 5a). As it was observed in experiments presented in Figure 3a, the transduction of CD34^+^ cells with MIG-210 induced a progressive expansion of transduced cells, which implied that at the end of the incubation period most cells (>90%) were EGFP^+^. Significantly, the ectopic expression of BRCA1 in MMC-treated MIG-210 transduced cells, not only increased the formation of BRCA1 foci, but also of FANCD2 foci (see Figure 5b and representative pictures in Figure 5c).

Our data thus show that *BCR/ABL* accounts for the impaired formation of BRCA1 foci in *BCR/ABL* expressing cells, and that this effect mediates an impairment in the generation of nuclear FANCD2 foci in these cells.

### The ectopic expression of BRCA1 reverts the generation of aberrant centrosomes induced by *BCR/ABL*

Because BRCA1 inhibition caused amplification and fragmentation of centrosomes in cells from mammary tissue[31], in the next set of experiments we aimed to investigate the role of BRCA1 in centrosome aberrations, characteristic of *BCR/ABL* cells[32]. To this aim, we investigated the presence of supernumerary centrosomes (more than 2 centrosomes per cell) in control CD34^+^ cells, as well as in *BCR/ABL* CD34^+^ cells, either re-transduced with a control Neo^r^ RV or with a BRCA1/Neo^r^ RV. The experimental protocol used in these experiments was similar to the one described in the immunofluorescence studies of Figure 5.

**Figure 6:**
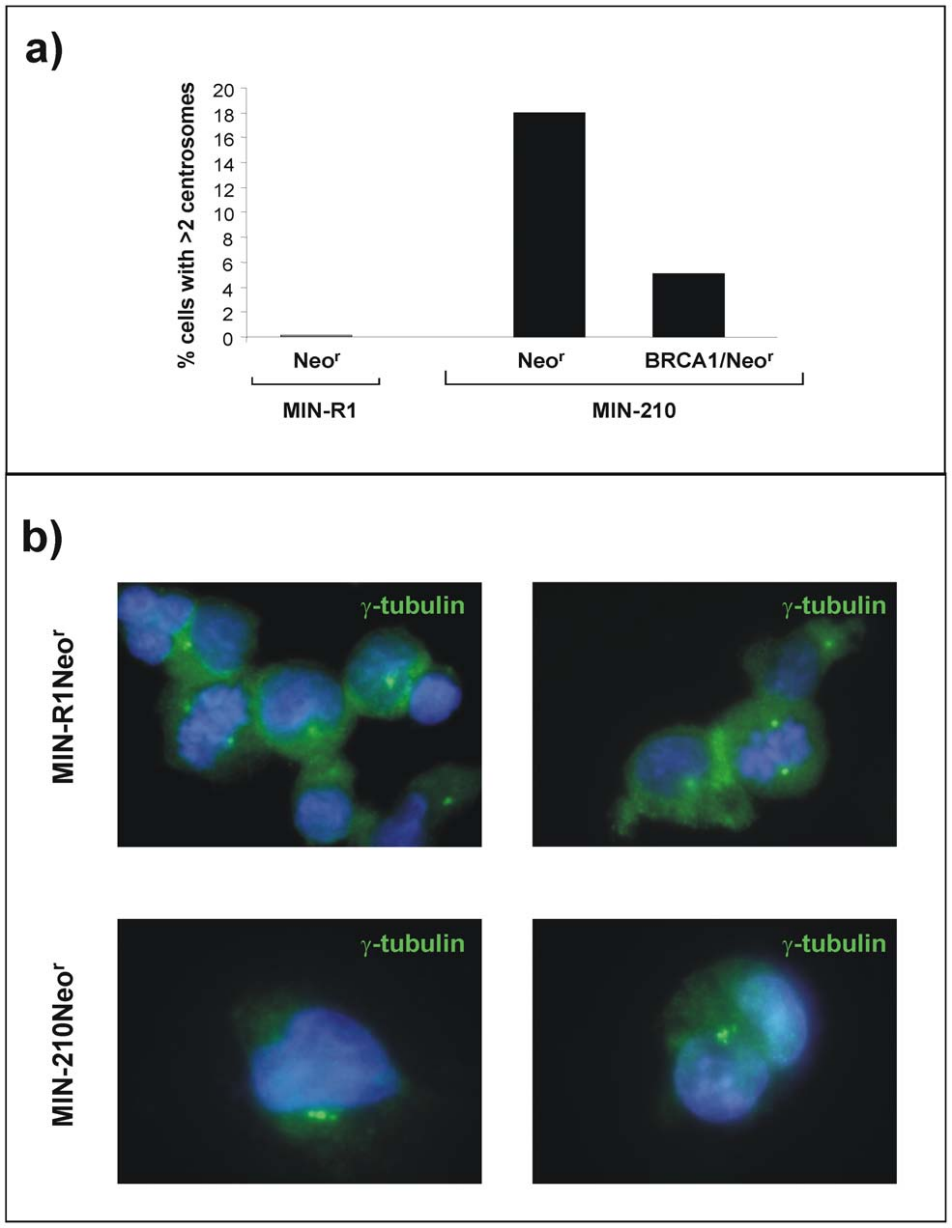
The ectopic expression of BRCA1 reverts the generation of aberrant centrosomes induced by *BCR/ABL*. a) Analysis of MIN-R1 or MIN-210-transduced cord blood CD34^+^cells with supernumerary centrosomes after re-infection with either Neor or BRCA1/Neor RVs. In all instances cells were exposed to 40 nM MMC prior to analysis. Data corresponding to one representative experiment is shown. b) Representative pictures corresponding to panel a) showing supernumerary centrosomes in MIN-210 *Neo^r^* compared to MIN-R1 *Neo^r^* cells. To identify centrosomes γ-tubulin antibody (green) was used. DAPI staining is shown in blue.

In contrast to control CD34+ cells, where only cells with one or two centrosomes were observed, the mere expression of the *BCR/ABL* induced multiple aberrant centrosomes in these cells as early as 9 days post-transduction (see Figure 6a and representative pictures in Figure 6b). Moreover, these experiments showed that the ectopic expression of BRCA1 in *BCR/ABL* cells reverted the generation of aberrant centrosomes induced by *BCR/ABL*. This observation demonstrates the role of disrupted pathways associated to BRCA1 down-regulation in the centrosomal instability of CML cells.

### The BCR/ABL-mediated interference of the FA/BRCA pathway does not compromise cell survival to DNA cross-linking drugs though induces chromosomal instability

**Figure 7:**
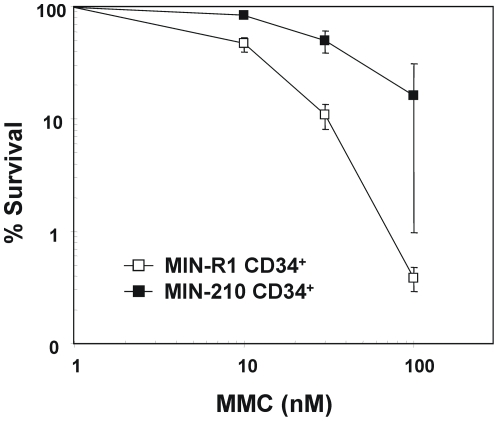
*BCR/ABL* induces mitomycin C resistance in cord blood progenitor cells. Cord blood CD34^+^ cells transduced with MIN-R1 or MIN-210 RVs were purified and cultured in methylcellulose plates with increasing concentrations of MMC. Fourteen days later the total number of CFCs was scored. The graphic represents mean ± s.e of survival data obtained from three independent experiments. The IC50 value of MMC corresponding to CFCs transduced with control and *BCR/ABL* vectors was, respectively: 11.78±1.25 and 51.79±18.24 nM.

Because defects in the FA/BRCA pathway may compromise the survival of *BCR/ABL* cells exposed to DNA cross-linking drugs[26] in the next set of experiments we investigated the sensitivity of *BCR/ABL* and control CD34^+^ cells to MMC. To this aim, CB CD34^+^ cells were transduced with the MIN-210 RV and the corresponding control MIN-R1 RV. Two days after transduction, cells were subjected to immunomagnetic cell sorting and cultured in methylcellulose with increasing concentrations of MMC. Fourteen days later, colonies were scored and MMC-survival curves determined[23]. As shown in Figure 7, MIN-210 transduced CD34^+^ cells were 5-fold more resistant to MMC compared to control MIN-R1 transduced cells (IC50: 51.79±18.24 nM and 11.78±1.25 nM MMC, respectively). These results contrast to the classical MMC-hypersensitivity observed in cells with a disrupted FA/BRCA pathway, indicating that other pathways promoting cell survival are up-regulated in *BCR/ABL* cells. This observation is consistent with previous data showing the ability of BCR/ABL to interfere with cellular apoptosis pathways[33], [34], [35].

**Figure 8:**
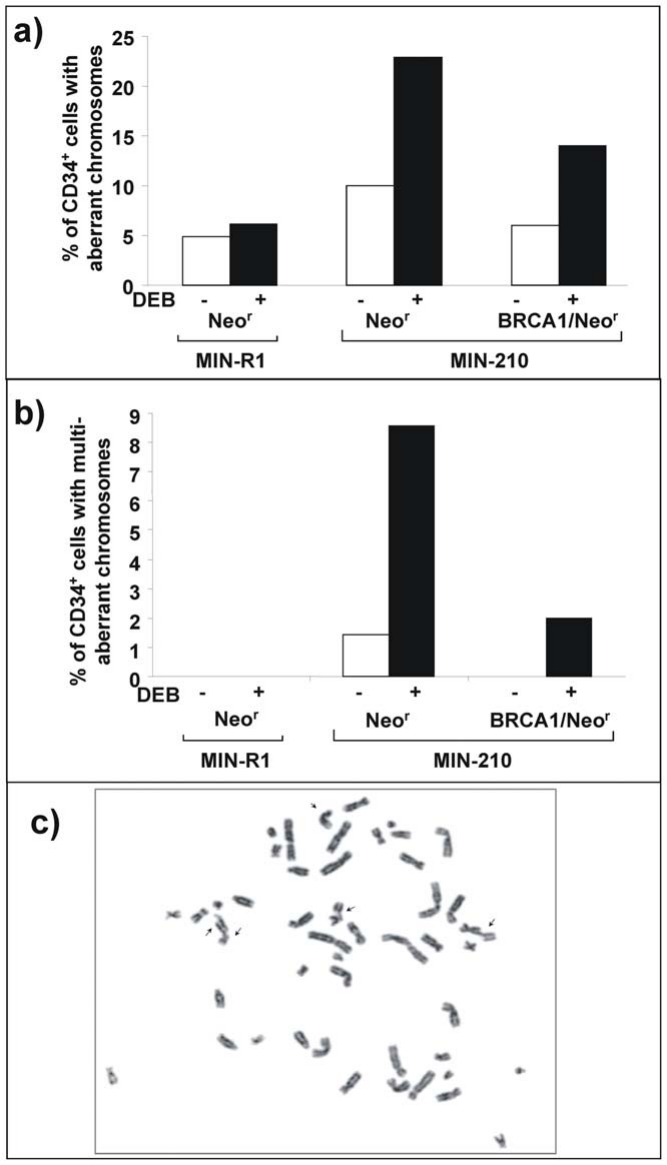
The ectopic expression of BRCA1 reverts the generation of chromosomal aberrations induced by *BCR/ABL*. a) Analysis of chromosomal aberrations in human cord blood CD34^+^ cells transduced with MIN-R1 or MIN-210 and re-infected with Neo^r^ or *BRCA1/neo^r^* RVs. Data corresponding to samples unexposed or exposed to DEB (0.1 µg/ml) are shown. b) Analysis of cells with multiple chromosomal aberrations in samples corresponding to panel a. Multiaberrant cells consisted on cells with two or more chromosomal breaks per cell. Data show the percentage of cells with aberrant and multiaberrant chromosomes, as deduced from the scoring of at least 50 metaphases. Pooled data obtained from two experiments with MIN RVs and one with MIG RVs are represented. c) Representative microphotograph of a multiaberrant metaphase *BCR/ABL* CD34^+^ Neo^r^ in the presence of DEB. Chromatid-type aberrations are shown with arrows.

Finally, because cells with an impaired FA/BRCA pathway are characterized by an increased chromatid-type chromosomal instability, particularly after exposure to DNA cross-linking drugs, we investigated the spontaneous and DEB-induced chromosomal instability of CD34^+^ cells previously transduced with the BCR/ABL RV (MIN-210) and its respective control (MIN-R1). Additionally, to test the influence of the ectopic expression of *BRCA1* on the chromosomal instability of *BCR/ABL* cells, *BCR/ABL*-transduced samples were re-transduced with control (*Neo^r^*) or *BRCA1* (*BRCA1/Neo^r^*) RVs, as described in materials and methods. In one experiment, chromosomal instability data was confirmed with MIG-R1 and MIG-210 RVs. Because similar data were obtained in this experiment, data in Figure 8 shows pooled results obtained with both vector families.

As shown in Figure 8a, a low proportion of control CD34^+^ cells (cells transduced with MIG-R1 or MIN-R1 RVs plus the control *Neo^r^* vector) either unexposed or DEB-exposed cells, contained chromosomal aberrations (5% and 7%, respectively). In no instance multiple chromosomal aberrations were observed in this control group, regardless that samples were exposed to DEB or not (Figure 8b). When CD34^+^ cells were transduced with *BCR/ABL* RVs (plus the control *Neo^r^* vector), the proportion of cells with chromosomal aberrations, specially of chromatid-type (see representative picture in Figure 8c), increased 2-fold in unexposed cells, and 3- fold in DEB-exposed cells, compared to control CD34^+^ cells (Figure 8a). Differences were even more marked when cells with multiple chromosomal aberrations were scored, mainly after DEB exposure. In this case, almost 10% of the metaphases contained two or more aberrant chromosomes (Figure 8b). Notably, the proportion of *BCR/ABL* cells with aberrant (Figure 8a) - and more markedly with multi-aberrant chromosomes (Figure 8b) - was reduced when these cells were re-transduced with the *BRCA1/Neo^r^* RV.

Taken together, these results show that the disruption of the FA/BRCA pathway in *BCR/ABL* cells mediates centrosomal amplification and chromosomal instability, and that this effect can be partially reverted by the ectopic expression of BRCA1.

## Discussion

Our study aims to offer new clues to understand the molecular pathways accounting for the genetic instability of CML cells. Our hypothesis that defects in the FA pathway may play a role in this process derive from previous studies showing the relevance of the FA pathway to control the genomic stability of the cell[18], [19] and also from observations showing genetic and epigenetic alterations of FA genes, both in inherited and acquired cancer[36], [37], [38], [39], [40], [41].

In our first experiments we investigated the ability of CML cells to generate FANCD2 nuclear foci, a central process in the FA pathway (see review in[20]), both during cell proliferation and after exposure to DNA cross-linking agents. Using Mo7e-p210 and CD34^+^ cells from CML patients, we observed that in contrast to normal cells, a very low proportion of cells harboring the *BCR/ABL* oncogene generated FANCD2 nuclear foci, even after treatment with MMC (Figure 1).

Because both the Mo7e-p210 cell line and also cells from CML patients may have accumulated secondary mutations that could account for their defective capacity to form FANCD2 foci, in subsequent experiments healthy hematopoietic progenitors consisting in CB CD34^+^ cells transduced with vectors expressing the *BCR/ABL* oncogene were used. Previous studies have shown that human CD34^+^ cells transduced with *BCR/ABL* vectors reproduce many of the characteristics seen in primary CML progenitors, facilitating the study of the molecular mechanisms involved in the transformation of hematopoietic precursors towards CML cells[42], [43]. Our studies with human CB CD34^+^ cells demonstrate that the mere transduction of these cells with *BCR/ABL* vectors is sufficient to inhibit the formation of FANCD2 foci, either in untreated or in MMC-treated cells (Figure 2). The relevance of the tyrosine kinase activity of BCR/ABL to inhibit the formation of FANCD2 foci was also demonstrated in these experiments by the observation that imatinib significantly restored the generation of FANCD2 foci in *BCR/ABL* cells.

Although FANCD2 monoubiquitination is required for the accumulation of FANCD2 in nuclear foci[27], our observations showing efficient FANCD2 monoubiquitination in CD34^+^ cells transduced with the MIG-210 vector (either exposed or not to MMC; Figure 3) demonstrate that p210 does not interfere with the upstream steps of the FA pathway.

In a recent report, Koptyra *et al* observed higher levels of FANCD2 monoubiquitination in cells from CML patients and also in *BCR/ABL*-transformed cells, compared to wild type cells, and proposed that this effect could play a role in *BCR/ABL* leukemogenesis[44]. Although we cannot rule out potential effects of BCR/ABL in up-modulating the monoubiquitination of FANCD2, we propose that the most relevant effect of this oncoprotein in the FA pathway is related to the inhibited translocation of FANCD2 to the chromatin. In this respect, different observations from other authors allowed us to hypothesize that one of the best candidates that may interfere with the translocation of FANCD2 to the nucleus of *BCR/ABL* cells was BRCA1. First, BRCA1 is post-transcriptionally down-regulated by p210[15]; second, while BRCA1 is not essential for FANCD2 monoubiquitination[28] it is required for FANCD2 binding to γH2AX at stalled replication forks[29] and for the subsequent formation of FANCD2 foci after DNA damage[27], [28]; and third, *BRCA1* ^−/−^ cells share with FA cells a chromosomal instability phenotype[45]. Additionally, because BRCA1 deficient cells have a defect in the G2/M checkpoint [45], our cell cycle studies showing that MMC-treated BCR/ABL cells are not arrested in G2/M - as it is characteristic of FA cells[26] - further suggest the role of BRCA1 in the interference of the FA pathway in these cells.

To clarify the mechanisms involved in the repression of BRCA1, and consequently in the impaired FANCD2 foci formation of CML cells, we were interested in further investigating the post-translational regulation of BRCA1 by the proteasome and the PI3K/AKT pathway, frequently activated in human cancer cells, including CML cells[46]. In this respect, data obtained in primary cells and in breast and ovarian cancer cell lines has shown that AKT1 represses BRCA1 foci formation[47], [48]. Strikingly, our results show that the inhibition of PI3K/AKT pathway with LY294002 restored not only BRCA1 but also FANCD2 foci in *BCR/ABL*-transduced CD34+ cells. The same effect was observed with the proteasome inhibitor, MG132, indicating that this molecule not only restores BRCA1 expression in BCR/ABL cells, as previously described[15], but also the formation of BRCA1 and FANCD2 foci in these cells. Finally, our data in *BCR/ABL* cells co-transduced with *BRCA1-* RVs (Figure 5) confirms that the ectopic expression of BRCA1 restores, at least in part, the inhibited formation of FANCD2 foci in *BCR/ABL* cells.

As it has been previously reported, centrosome amplification occurs frequently in all types of cancer and this correlates with the malignant progression of the disease[49]. As it is the case in BRCA1-deficient cells[45], centrosome aberrations and aneuploidy are also common features of CML. In fact, previous data have shown that centrosome abnormalities correlated with the CML disease stage and preceded chromosomal aberrations in primary cells from CML patients[32]. By means of the ectopic expression of BRCA1 we show the involvement of BRCA1 in centrosomal aberrations observed in CD34^+^ cells soon after their transduction with BCR/ABL RVs, supporting the hypothesis that this phenotype constitutes an early event in the transformation of CML cells.

The generation of centrosomal abnormalities by *BCR/ABL* is consistent with the chromosomal instability of these cells, an observation that is particularly evident after exposure to different DNA damaging agents, including oxygen species, ionizing radiation, or etoposide[10], [12]. In this respect, our study shows for the first time the chromosomal instability of *BCR/ABL*-transduced CD34^+^ cells exposed to a DNA cross-linking drug, DEB, classically used for the diagnosis of FA[50]. Strikingly, our results show that BCR/ABL confers a survival advantage, while mediates chromosomal instability to DNA cross-linking agents. The observation that BCR/ABL induces a survival advantage to MMC is, however, not surprising considering that this oncoprotein interferes with several pathways activating apoptosis[33], [34], [35]. In this respect, early studies showed that BCR/ABL mediates protection from DNA-damaged apoptosis in a dose-dependent manner[51], due to the capacity of the oncoprotein to regulate the expression and/or the activity of several pro- and anti-apoptotic factors signaling through the STAT5[21], PI3K/AKT[52] and RAS[53] pathways.

Finally, of particular significance was the observation that the ectopic expression of *BRCA1* in *BCR/ABL* cells markedly decreased the number of cells with aberrant, and more significantly with multi-aberrant chromosomes. Because *BRCA1* vectors also restored the formation of FANCD2 foci in *BCR/ABL* CD34^+^ cells, our results add new insights to data previously obtained by Deutsch *et al* [15] who showed a down-regulated expression of *BRCA1* in *BCR/ABL* hematopoietic cells. In particular, our data strongly suggest that this effect interferes with the translocation of FANCD2 to sites of DNA damage, thus compromising the genomic stability of *BCR/ABL* cells.

Taken together, data obtained in this study allow us to propose that the malignant phenotype conferred by *BCR/ABL* should be at least in part related to its capacity to interfere both with downstream steps of the FA/BRCA pathway and also with other pathways with a role in apoptosis[33], [34], [35]. As a result of the simultaneous interference of these pathways, a survival advantage should occur in *BCR/ABL* hematopietic progenitors harboring genomic alterations which may be not compatible with the survival of untransformed cells. We therefore propose that a defective FA/BRCA pathway may contribute to the genomic instability of CML cells, thus promoting the accumulation of mutations during the progress from a chronic phase towards blast crisis.

## Supporting Information

Figure S1 **Cell cycle analysis of cord blood CD34^+^ cells transduced with MIG-R1 and MIG-210 retroviral vectors.** Histograms show cell cycle distributions 7 days after transduction of healthy cord blood CD34^+^ cells with MIG-R1 or MIG-210, and exposed to 40 nM MMC (see schematic protocol in Figure 2a). At this time, more than 90% of cells exposed to the MIG-210 RV were EGFP^+^.
(TIF)

Figure S2 **Analysis of the generation of double strand breaks in cord blood CD34^+^ cells transduced with MIG-R1 and MIG-210 retroviral vectors.** The Figure shows the proportion of MIG-R1 (white bars) and MIG-210 (grey bars) transduced CD34^+^ cells with nuclear γ-H2AX foci, both in untreated and in MMC treated (40 nM, 16 h) cells. Data from a representative experiment is shown. Representative pictures of cells with γ-H2AX foci are also shown.
(TIF)
