# Supplementary figures and images for: Bcr/Abl Interferes with the Fanconi Anemia/BRCA Pathway: Implications in the Chromosomal Instability of Chronic Myeloid Leukemia Cells
Source: PLoS One. 2010 Dec 28;5(12):e15525. doi: 10.1371/journal.pone.0015525 (PMC3011007; doi:10.1371/journal.pone.0015525)

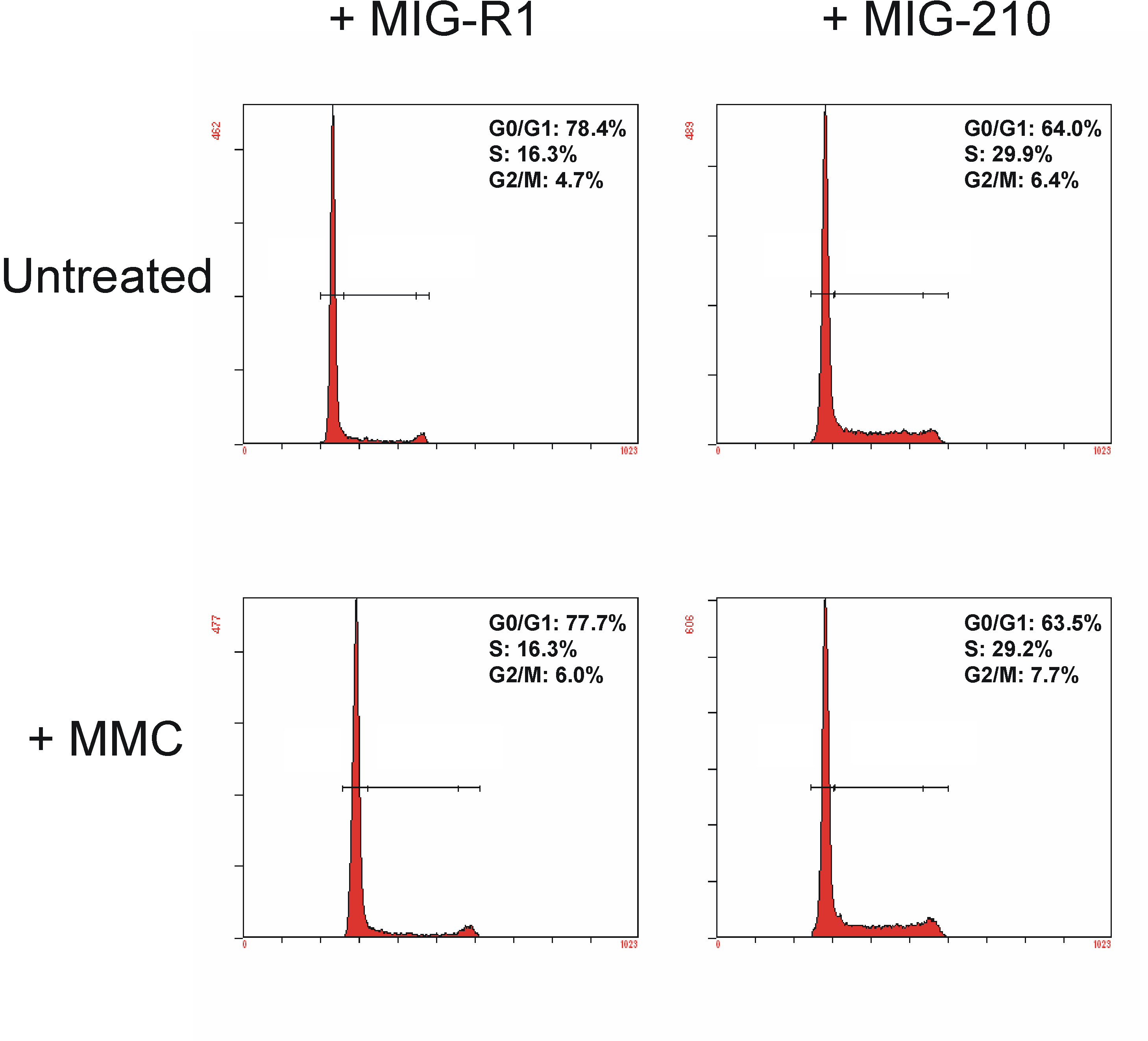

Supplement: Figure S1 — Cell cycle analysis of cord blood CD34+ cells transduced with MIG-R1 and MIG-210 retroviral vectors. Histograms show cell cycle distributions 7 days after transduction of healthy cord blood CD34+ cells with MIG-R1 or MIG-210, and exposed to 40 nM MMC (see schematic protocol in Figure 2a). At this time, more than 90% of cells exposed to the MIG-210 RV were EGFP+. (TIF) [file pone.0015525.s001.tif]

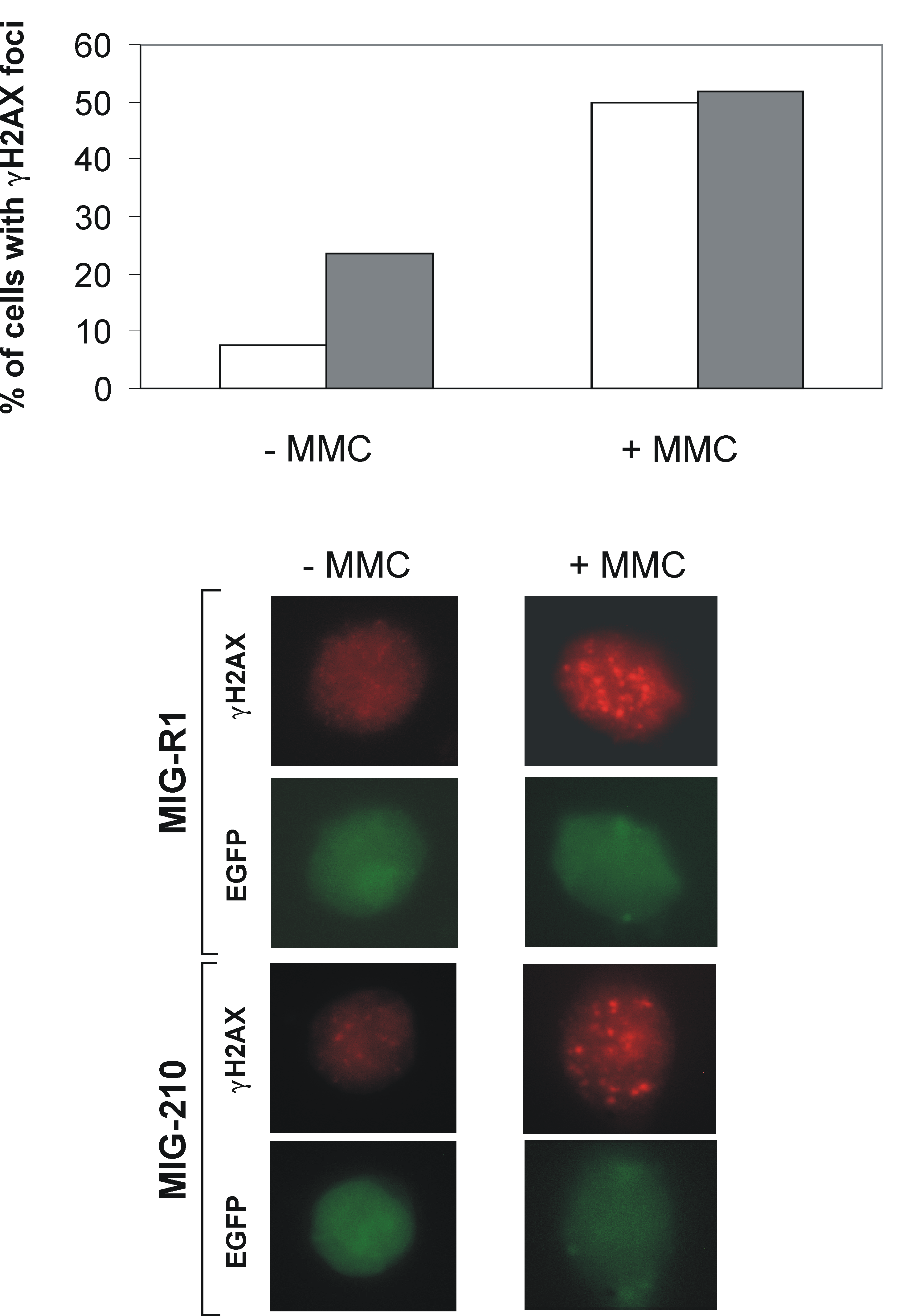

Supplement: Figure S2 — Analysis of the generation of double strand breaks in cord blood CD34+ cells transduced with MIG-R1 and MIG-210 retroviral vectors. The Figure shows the proportion of MIG-R1 (white bars) and MIG-210 (grey bars) transduced CD34+ cells with nuclear γ-H2AX foci, both in untreated and in MMC treated (40 nM, 16 h) cells. Data from a representative experiment is shown. Representative pictures of cells with γ-H2AX foci are also shown. (TIF) [file pone.0015525.s002.tif]
